# Discovery and validation of miR-452 as an effective biomarker for acute kidney injury in sepsis

**DOI:** 10.7150/thno.50093

**Published:** 2020-10-25

**Authors:** Zhiwen Liu, Danyi Yang, Jingli Gao, Xiaohong Xiang, Xiaoru Hu, Siyao Li, Wenwen Wu, Juan Cai, Chengyuan Tang, Dongshan Zhang, Zheng Dong

**Affiliations:** 1 Department of Nephrology, The Second Xiangya Hospital at Central South University, Changsha, Hunan, China.; 2 Department of Emergency Medicine, The Second Xiangya Hospital at Central South University, Changsha, Hunan, China.

**Keywords:** acute kidney injury, biomarker, microRNA, NF-κB, sepsis

## Abstract

**Rationale:** Sepsis is the cause of nearly half of acute kidney injury (AKI) and, unfortunately, AKI in sepsis is associated with unacceptably high rates of mortality. Early detection of AKI would guide the timely intervention and care of sepsis patients. Currently, NephroCheck, based on urinary [TIMP2]*[IGFBP7], is the only FDA approved test for early detection of AKI, which has a relatively low sensitivity for sepsis patients.

**Methods:** *In vitro*, BUMPT (Boston University mouse proximal tubular cell line) cells were treated with lipopolysaccharides (LPS). *In vivo*, sepsis was induced in mice by LPS injection or cecal ligation and puncture (CLP). To validate the biomarker potential of miR-452, serum and urinary samples were collected from 47 sepsis patients with AKI, 50 patients without AKI, and 10 healthy subjects.

**Results:** miR-452 was induced in renal tubular cells in septic AKI, and the induction was shown to be mediated by NF-κB. Notably, serum and urinary miR-452 increased early in septic mice following LPS or CLP treatment, prior to detectable renal dysfunction or tissue damage. Sepsis patients with AKI had significantly higher levels of serum and urinary miR-452 than the patients without AKI. Spearman's test demonstrated a remarkable positive correlation between urinary miR-452 and serum creatinine in sepsis patients (r=0.8269). The area under the receiver operating characteristic curve (AUC) was 0.8985 for urinary miR-452. Logistic regression analysis showed a striking 72.48-fold increase of AKI risk for every 1-fold increase of urinary miR-452 in sepsis patients. The sensitivity of urinary miR-452 for AKI detection in sepsis patients reached 87.23%, which was notably higher than the 61.54% achieved by urinary [TIMP2]*[IGFBP7], while the specificity of urinary miR-452 (78.00%) was slightly lower than that of [TIMP2]*[IGFBP7] (87.18%).

**Conclusions:** miR-452 is induced via NF-κB in renal tubular cells in septic AKI. The increase of miR-452, especially that in urine, may be an effective biomarker for early detection of AKI in sepsis patients.

## Introduction

Acute kidney injury (AKI) is major kidney disease characterized by a rapid decline of renal function in a short period of time. Clinically, the main causes of AKI include sepsis, renal ischemia-reperfusion, and exposure to nephrotoxins, among which sepsis accounts for nearly half of all AKI cases 1-4. Unfortunately, AKI in sepsis is associated with a very poor prognosis 1, 2, 5-10 and the mortality rate in sepsis patients with AKI is 3-5 fold higher than the sepsis patients without AKI 11. Early detection of AKI in sepsis would enable timely intervention and guide the treatment and care for these patients 1-3, 6, 7.

Currently, serum creatinine and urine output are commonly used for AKI diagnosis and staging. However, changes in serum creatinine and urine output occur relatively late after actual kidney injury and, therefore, cannot provide an early or timely detection of AKI. Moreover, serum creatinine and urine output are affected by a multitude of factors, such as dehydration, dietary protein intake, muscle mass, diuretics, *etc.* 12. In recent years, a number of molecular biomarkers for AKI have been discovered, including kidney injury molecule-1 (KIM-1), neutrophil gelatinase-associated lipocalin (NGAL), interleukin-18 (IL-18), liver fatty acid-binding protein (L-FABP), tissue inhibitor of metalloproteinase 2 (TIMP2), insulin-like growth factor binding protein 7 (IGFBP7) and cystatin C, to just name a few 13-16. Among them, the arithmetic product of the concentrations of urinary TIMP2 and IGFBP7 ([TIMP2]*[IGFBP7]) demonstrated high specificity and sensitivity as a biomarker for AKI in multiple clinical studies 17-19. In 2014, NephroCheck®, based on urinary [TIMP-2]*[IGFBP7], was approved by U.S. Food and Drug Administration (FDA) for marketing as a biomarker test for early detection of AKI. Nonetheless, the increase of urinary TIMP2 and IGFBP7 in AKI mainly depends on renal proximal tubule injury, raising the concern that mild tubular injury in septic AKI would reduce the sensitivity of [TIMP2]*[IGFBP7] in early AKI detection in sepsis patients 20.

MicroRNAs (miRNAs) are short, non-coding RNA molecules of ~22 nucleotides that are produced endogenously to regulate target gene expression. Interesting, some miRNAs are released into extracellular space and become detectable in blood, urine and other body fluids, representing novel diagnostic biomarkers for human diseases. Compared to protein biomarkers, miRNAs are more stable in body fluids 21, 22. In addition, sequence-based qPCR amplification enables the measurement of very low amounts of miRNAs in a small volume of body fluids with high specificity 23-25. In kidneys, miRNAs have been implicated in the pathogenesis of various renal diseases including AKI, and the expression changes in specific miRNAs were suggested to have diagnostic values for AKI 23, 26, 27. For example, sepsis patients with AKI had higher levels of serum miR-4321 and miR-4270 than sepsis patients without AKI 28. However, miRNA biomarkers with high sensitivity and specificity for early detection of AKI in sepsis patients are currently unavailable. In the present study, we have demonstrated miR-452 induction in experimental models of septic AKI, and have further validated urinary miR-452 as an effective biomarker for early detection of AKI in sepsis patients.

## Methods

### Study approval

Animal experiments were conducted in accordance with a protocol approved by the Institutional Animal Care and Use Committee of the Second Xiangya Hospital of Central South University and the NIH Guide for the Care and Use of Laboratory Animals. The human study was approved by the Ethics Committee of The Second Xiangya Hospital of Central South University (Approved protocol number: 2013001). All participants prior to inclusion in the study were recruited with written informed consent.

### Antibodies and special reagents

The antibodies used in the present study were from the following sources: anti-p65 (catalog number: 8242), anti-p-p65 (3033), anti-GAPDH (5174) from Cell Signaling Technology (Boston MA); all secondary antibodies for immunoblot analysis from Thermo Fisher Scientific (Waltham Massachusetts); Alexa Fluor 594-conjugated goat anti-rabbit IgG (ab150080) from Abcam (Cambridge, UK). Special reagents were purchased from the following sources: lipopolysaccharides (Sigma, St. Louis, MO), TPCA-1 (APExBIO, Houston, TX), digoxigenin-labeled mmu-miR-452 LNA probe (Servicebio, Wuhan, China), Fluorescence *in situ* hybridization Kit (Servicebio, Wuhan, China), anti-DIG-HRP (Jackson, MS), human/mouse tissue inhibitor of metalloproteinase 2 (TIMP2) and human/mouse Insulin-like growth factor binding protein 7 (IGFBP7) ELISA Kits (CUSABIO, Wuhan, China).

### Mouse model of septic AKI

Male C57BL/6 mice (8~10 weeks) were purchased from Hunan Slack King Experimental Animal Company (Changsha, China). The animals were maintained in pathogen-free condition under a 12-hour light/dark cycle with free access to food and water. AKI was induced by lipopolysaccharides (LPS) injection or by cecal ligation and puncture (CLP). For LPS-induced-AKI, one dose of LPS (10 mg/Kg body weight) was intraperitoneally injected 29. Control mice were given saline. For CLP-induced AKI, CLP surgery was performed as previously described 30. Briefly, after anaesthesia with 60 mg/Kg pentobarbital, mice were placed on Homeothermic Blanket Control Unit (507220F, Harvard Apparatus) to maintain body temperature at 36.5 °C. A small longitudinal midline incision was made to expose the cecum which was then ligated at 1 cm away from the blind-ending. A single through-and-through puncture midway between the ligation and the tip of the cecum in a mesenteric-to-antimesenteric direction was made to perforate the cecum. After removing the needle, a small droplet of feces was extruded from both penetration holes to ensure patency. Mice underwent the same operation but without cecal ligation and puncture were used as sham control 30. The mice were sacrificed at 4 h and 12 h after LPS injection or CLP to collect kidney tissues, blood, and urine for analysis.

### Cells and LPS treatment

The Boston University mouse proximal tubular cell line (BUMPT) was originally provided by Drs. Wilfred Lieberthal and John Schwartz at Boston University School of Medicine (Boston, MA). The cells were cultured in DMEM medium containing 10% fetal bovine serum (FBS). For LPS treatment, BUMPT cells at a 50-60% confluency were maintained in DMEM medium containing 0.2% FBS for 24 h. The cells were then changed to DMEM medium containing 0.2% FBS plus 10 μg/mL LPS for another 8 h. For TPCA-1 treatment, TPCA-1 was added concurrently with LPS at the final concentration of 100 μM 31.

### MicroRNA microarray analysis

MicroRNA microarray was conducted as described in our previous work 32, 33. Briefly, total RNAs were extracted from kidney cortex of mice with or without LPS treatment. The RNA samples were reverse transcribed using the TaqMan MicroRNA Reverse Transcription Kit (Applied Biosystems, Foster City, CA). The product from each reverse transcription reaction was pre-amplified and then the microRNA expression was profiled with TaqMan Rodent MicroRNA array card A v2.0 following the manufacturer's protocol. The global normalization process included the subtraction of the mean CT value of the reference set from the CT value of each microRNA of the same sample. Quantification of each sample was shown as 2^-ΔΔCt^ values.

### Quantitative real time-PCR

Total RNAs from cells and kidney tissues were isolated with the mirVana kit (Ambion, Austin, TX). Total RNAs from urine and serum were isolated with the miRNeasy Serum/Serum Kit (QIAGEN, Germantown, MD). 50 ng of total RNAs from each sample were reversely transcribed into cDNA by using the microRNA Reverse Transcription kit (Applied Biosystems, Beverly, MA). Real-time quantitative PCR was performed by using the Taqman microRNA assay kit (Applied Biosystems). We used sno202 as an endogenous control miRNA when determining miR-452 levels in kidney tissues and used cel-39 an exogenous control miRNA when evaluating miR-452 levels in serum and urine. The cycle thresholds (Ct) for miR-452 and the reference miRNA from each individual sample were determined. For calculation, ΔCt = Ct_miR-452_ - Ct_reference gene_, and ΔΔCt =Δ Ct _sepsis_ -Δ Ct _control_. The fold of miR-452 expression in sepsis group over control group is 2^-ΔΔCt^ 31, 34.

### Chromatin immunoprecipitation

Chromatin immunoprecipitation (Chip) assay was conducted as described in our recent work 34. Briefly, cells were cross-linked with 1% formaldehyde and then lysed. The cell lysate was sonicated to fragment the DNA and then centrifuged to collect the supernatant containing DNA. Equal amounts of DNA from different samples were incubated with equal amount of anti-p65 antibody for immunoprecipitation. The resultant immunoprecipitates were subjected to real-time qPCR for amplification of the putative NF-κB binding sequence using specific primers: Forward: 5'-TGCCACGATCAAGAGGTCAG-3'; Reverse: 5'-CCTTCAGTTAATGTAAGCCAGGT-3'. The value of real-time qPCR was normalized with input DNA for comparison.

### Fluorescence *in situ* hybridization

Fluorescence *in situ* hybridization (FISH) analysis of miR-452 in kidney tissues were performed as described in our recent work 32, 34. Briefly, paraffin-embedded kidney sections were subjected to deparaffinization, hydration, permeabilization with 20 μg/mL proteinase K, and incubation with pre-hybridization solution at 78 °C for 1 h. The tissues sections were then exposed to digoxigenin-labeled mmu-miR-452 LNA probe (Servicebio, Wuhan, China) at 37 °C overnight. After incubation with 5% bovine serum albumin (BSA) to reduce unspecific staining, the tissue sections were incubated with anti-digoxigenin-HRP at 37 °C for 1 h, followed by exposure to Cy3-TSA. To show cell nuclei, tissue sections were also stained with DAPI.

### Immunoblot analysis

Cells or kidney tissues were lysed with 2% SDS buffer containing protease inhibitor cocktail (Sigma-Aldrich, P8340). Protein concentration was determined with BCA reagent (Thermo Fisher Scientific). Equal amounts of proteins from different samples were separated on SDS-polyacrylamide gels and transferred onto Polyvinylidene difluoride membrane. The membrane was incubated with 5% fat-free milk to reduce unspecific signals, followed by incubation with primary antibodies and horseradish peroxidase-conjugated secondary antibodies. Antigen-antibody complex was revealed with an enhanced chemiluminescence kit (Thermo Fisher Scientific). GAPDH was used as an internal control to monitor protein loading and transferring.

### Renal morphological studies and immunofluorescence

For renal histology, paraffin-embedded kidney tissue sections of 4 µm thickness were stained with hematoxylin-eosin (HE). For immunofluorescence (IF), paraffin-embedded kidney sections were sequentially subjected to deparaffinization, hydration, and antigen retrieval by incubation with 0.1 M sodium citrate, PH 6.0 at 100 °C. The tissue sections were then incubated with 3% H2O2, 5% normal donkey serum and then 0.1% Triton X-100 to reduce non-specific binding. After that, the tissue sections were exposed to 1:100 anti-p-p65 antibody at 4 °C overnight followed by exposure to Alexa Fluor 594-conjugated secondary antibody for 1 h at room temperature. Sections were finally counterstained with DAPI (Sigma-Aldrich, D9542) and examined by laser scanning confocal microscopy. For quantification, 10-20 fields were randomly selected from each tissue section to assess the number of positive cells per mm^2^.

### Biochemical analysis of blood and urine

Serum was collected from blood samples after clotting and centrifugation. Serum creatinine and blood urea nitrogen (BUN) were measured to indicate renal dysfunction with reagents from BioAssay Systems (Hayward, CA). Urinary TIMP2 and IGFBP7 were measured by using an ELISA Kit according to the manufacturer's instruction.

### Human patients and sample collection

A total of 97 sepsis patients and 10 healthy people were recruited with written informed consent. Sepsis was diagnosed according to the International Sepsis Definition Conference criteria (Figure S1) 35. The patients were divided into two groups: 1) sepsis patients with AKI or septic AKI patients (n = 47) who developed AKI during the first 7-day observation period; and 2) sepsis patients without AKI or non-AKI sepsis patient (n = 50). AKI was analyzed and staged according to the KDIGO criteria (Table S1) 36. Urine and blood samples were collected and centrifuged at 3000 rpm for 10 min to remove cells and cellular debris. The supernatants were stored at -80 °C for future analysis. The clinical data of the patients (such as serum creatinine) were obtained from the Case Management System of the hospital.

### Statistics

Statistical differences between two groups were analyzed by student's t-test, and differences in multiple groups were determined by ANOVA analysis. Receiver operating characteristic (ROC) analysis and Logistic Regression were applied to evaluate the efficiency (sensitivity and specificity) of diagnostic indicators. Pearson's correlation analysis was performed to evaluate the strength of relationship between two quantitative variables. Data are expressed as the means ± SD. *P <* 0.05 was considered significant. GraphPad Prism 7.0 and SPSS 22.0 were used for statistical analysis.

## Results

### miR-452 is induced in renal tubules in LPS-induced AKI

**Figure 1:**
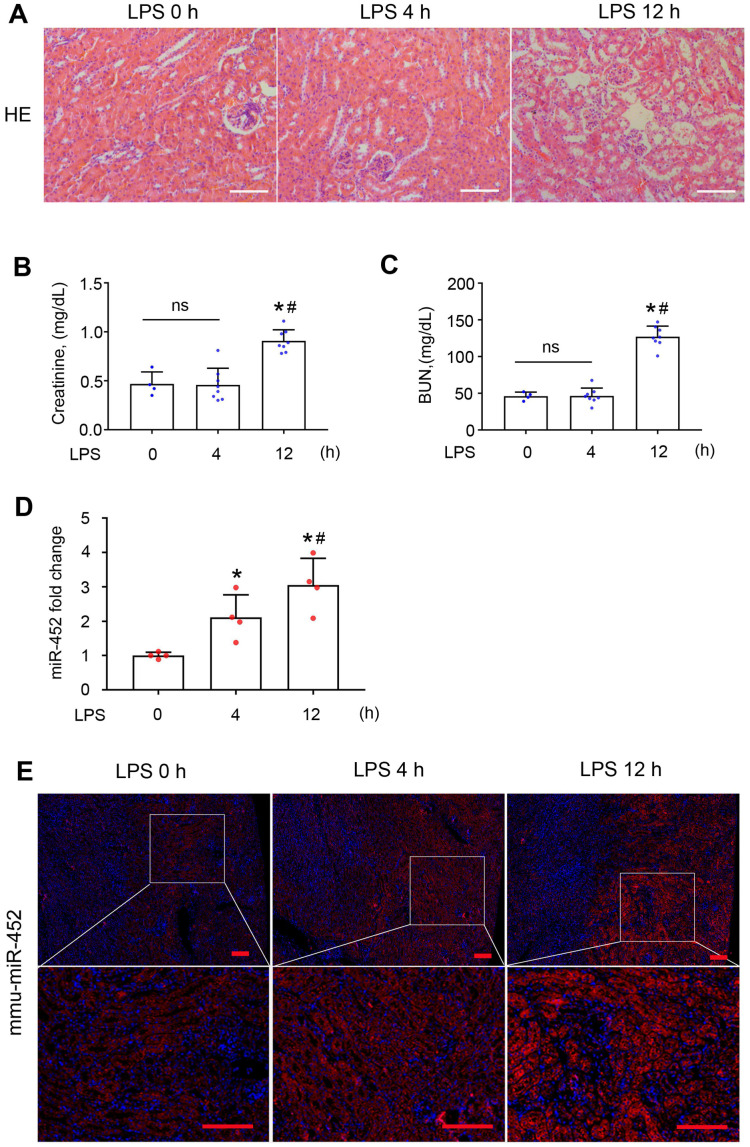
** miR-452 is induced in renal tubular cells in LPS-induced AKI.** Male C57BL/6 mice were injected with 10 mg/Kg LPS or saline (Control) to collect blood, urine, and kidney tissues at indicated time points. **(A)** Renal histology showing tissue damage at 12 h (but not 4 h) of LPS treatment. Scale bar: 100 µm. **(B)** Serum creatinine increase at 12 h (but not 4 h) of LPS treatment. Values are expressed as mean ± SD (n = 4 for each control group; n = 8 for each LPS treatment group), *P < 0.05 vs. Control (LPS 0 h), ^#^ *P <* 0.05 vs. LPS 4 h. **(C)** Increase of blood urea nitrogen (BUN) at 12 h (but not 4 h) of LPS treatment. Values are expressed as mean ± SD (n = 4 for each control group; n = 8 for each LPS treatment group), *P < 0.05 vs. Control (LPS 0 h), ^#^ *P <* 0.05 vs. LPS 4 h. **(D)** LPS-induced miR-452 up-regulation in kidney tissues in mice. miR-452 is normalized to the level of sno202 (internal control) to determine the ratios. The ratios of control mice were arbitrarily set as 1. Values are expressed as mean ± SD (n = 4), *P < 0.05 vs. control mice, ^#^ *P <* 0.05 vs. LPS 4 h. **(E)** FISH analysis showing miR-452 induction by LPS in kidney tubule cells. Lower panels are enlarged images of the selected areas in upper panels. Scale bar: 100 µm.

To identify the specific miRNAs altered in septic AKI, we conducted microarray analysis of kidney tissues from LPS-treated mice and control mice. LPS did not induce overt kidney tissue damage or increases in serum creatinine (Scr) and BUN at 4 h, but at 12 h there were noticeable tubular changes associated with significant rises in both Scr and BUN (Figure 1A-C). In microarray analysis, miR-452 showed increased expression at both time-points after LPS treatment. Quantitative real-time PCR (qRT-PCR) analysis confirmed the induction of miR-452 after LPS treatment (Figure 1D). Fluorescence *in situ* hybridization (FISH) further localized miR-452 induction mainly in tubular cells in renal cortex and outer medulla (Figure 1E). In addition, miR-452 was induced by LPS in cultured BUMPT cells, a mouse proximal tubular cell line (Figure S2).

### NF-κB mediates miR-452 induction during LPS-induced AKI

**Figure 2:**
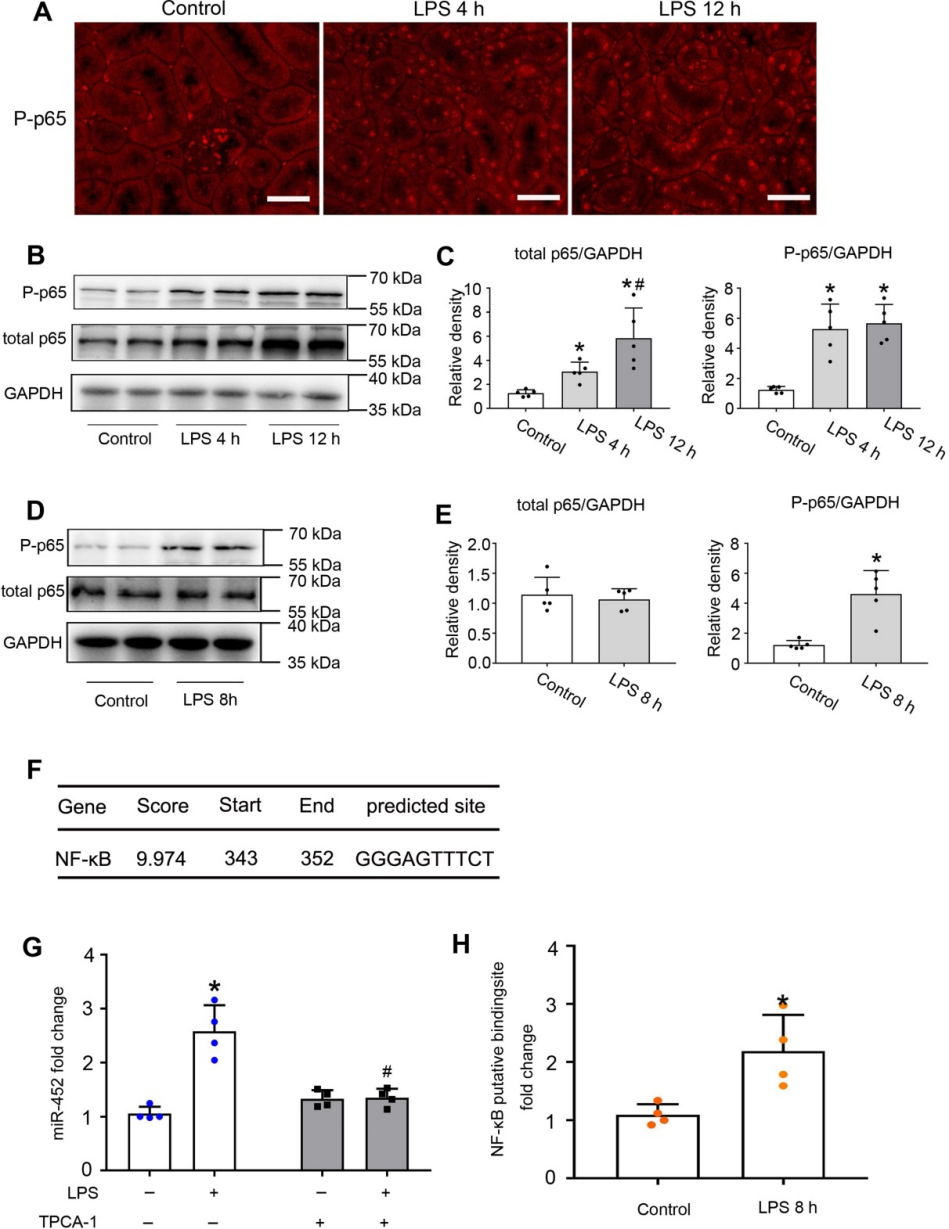
** NF-κB mediates miR-452 induction during LPS-induced AKI in mice**. Male C57BL/6 mice were injected with 10 mg/Kg LPS or saline as control to collect kidney tissues at indicated time points. (A) Immunofluorescence of p-p65 showing nuclear accumulation of p-p65 in renal tubule cells in LPS-treated mice. Scale bar: 50 µm. (B) Immunoblots showing the increases of p-p65 and p65 in kidney tissues in LPS-treated mice. GAPDH was used as internal control. (C) Densitometry analysis of total p65 or p-p65 levels in kidney tissues. Values are the mean ± SD (n = 5), *P < 0.05 vs. control, # *P <* 0.05 vs. LPS 4 h. (D) Immunoblots showing p-p65 induction by LPS in BUMPT cells. BUMPT cells were exposed to 10 µg/mL LPS for 8 h to collect whole cell lysate for immunoblot analysis. GAPDH was used as loading control. (E) Densitometry analysis of total p65 or p-p65 in BUMPT cells subjected LPS treatment. Values are the mean ± SD (n = 5), *P < 0.05 vs. Control. (F) Predicted NF-κB binding site in mouse miR-452 gene promoter. (G) Inhibition of LPS-induced miR-452 expression by TPCA-1. BUMPT cells were treated with LPS for 8 h with or without 100 µM TPCA-1. miR-452 was normalized to the level of sno202 to determine the ratios. The ratios of control were arbitrarily set as 1. Data are expressed as mean ±SD (n = 4), *P < 0.05 versus control, #*P <* 0.05 vs. LPS treatment group. (H) ChIP analysis showing LPS-induced NF-κB binding to miR-452 promoter. BUMPT cells were treated with or without LPS for 8 h to collect the chromatin for immunoprecipitation with a specific anti-p65 antibody. The immunoprecipitated samples were subjected to qRT-PCR analysis of the NF-κB binding sequence in miR-452 promoter. Data are expressed as mean ± SD (n = 4), *P < 0.05 versus control.

Activation of NF-κB has been reported in experimental models of septic AKI 37. In mammalian cells, there are five NF-κB family proteins, including RelA/p65, RelB, c-Rel, p50 and p52 38, 39. We detected the accumulation of phosphorylated p65 (p-p65) in the nucleus of renal tubular cells by immunofluorescence staining (Figure 2A). We further confirmed the induction of total and phosphorylated p65 by LPS in kidneys by immunoblot analysis (Figure 2B-C). Consistently, LPS induced p-p65 in cultured BUMPT cells (Figure 2D-E). Bioinformatics analysis predicted a potential NF-κB binding site at the gene promoter of mouse miR-452 (Figure 2F). With these results, we hypothesized that NF-κB might mediate miR-452 induction during LPS treatment via binding and activating it gene promoter. To test this possibility, we first examined the effect of TPCA-1, a commonly used inhibitor of NF-κB signaling. As shown in Figure 2G, LPS increased miR-452 expression in BUMPT cells, which was abolished by TPCA-1. Moreover, LPS induced a more than two-fold increase in the binding of p65/NF-κB to miR-452 gene promoter in ChIP assay (Figure 2H). Collectively, these results suggest that NF-KB mediates miR-452 induction during septic AKI.

### Serum and urinary miR-452 increases before renal dysfunction and tissue damage in both LPS and CLP-induced AKI in mice

**Figure 3:**
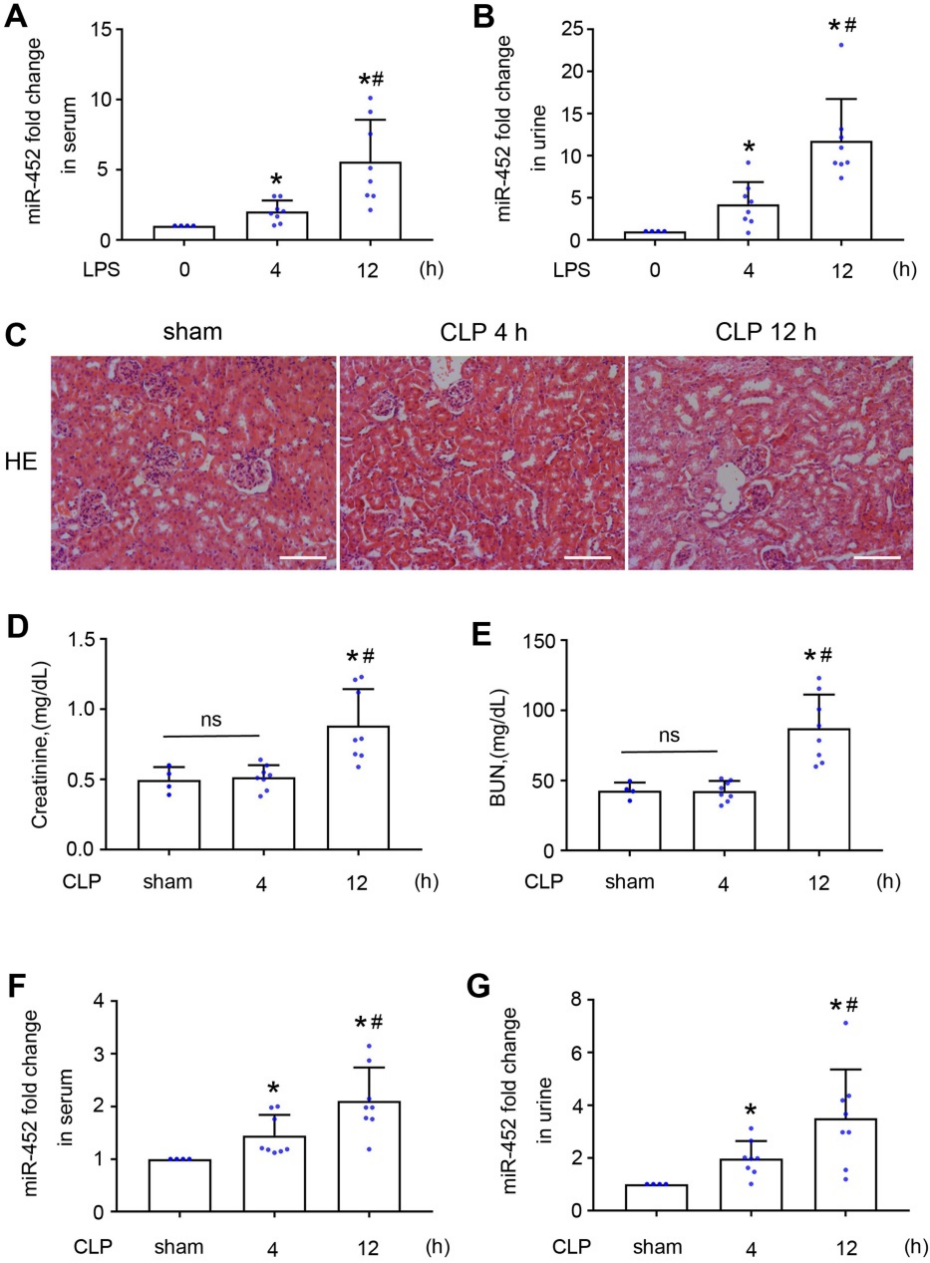
** Serum and urinary miR-452 increase before renal dysfunction and tissue damage in both LPS and CLP-induced AKI.** (A, B) Male C57BL/6 mice were injected with 10 mg/Kg LPS (or saline as control) to collect blood and urine samples at indicated time points for qPCR measurement of miR-452. All quantitative data are mean ± SD (n = 4 for each control group; n = 8 for each LPS treatment group), *P < 0.05 vs. LPS 0 h, #*P <* 0.05 vs. LPS 4 h. (C - G) Male C57BL/6 mice were subjected CLP or sham-operation to collect blood, urine, and kidney tissues at indicated time points. (C) Renal histology showing tissue damage at 12 h (but not 4 h) of CLP. Scale bar: 100 µm. (D) Serum creatinine increase at 12 h (but not 4 h) of CLP. (E) Increase of blood urea nitrogcen (BUN) at 12 h (but not 4 h) of CLP. (F) Increase in serum miR-452 at 4 and 12 h of CLP. (G) Increase in urinary miR-452 at 4 and 12 h of CLP. miR-452 was normalized with the level of added cel-miR-39 (exogenous control) in the same samples to determine the ratios, while the ratios of control were arbitrarily set as 1. All quantitative data are mean ± SD (n = 4 for sham control group; n = 8 for each CLP treatment group), *P < 0.05 vs. sham control, #*P <* 0.05 vs. CLP 4 h.

The observation of miR-452 induction at 4 h after LPS treatment in the absence of detectable kidney injury (Figure 1) suggested its potential as a biomarker for early detection of kidney injury. We therefore analyzed serum and urinary miR-452 in this model. As shown in Figure 3A-B, LPS induced significant miR-452 increases in both serum and urine samples at 4 h after LPS treatment and the increases became larger at 12 h. To extend this observation, we examined CLP-induced AKI, a model of sepsis-associated AKI in patients. Histological and biochemical analysis did not show obvious renal dysfunction or tissue damage at 4 h after CLP, while noticeable kidney injury was detected at 12 h (Figure 3C-E). However, significant increases of serum and urinary miR-452 were detected at 4 h after CLP, and the increase continued at 12 h (Figure 3F-G). Together, these results suggest that serum and urinary miR-452 may be a potential biomarker for early detection of septic AKI.

### Sepsis patients with AKI have higher levels of urinary and serum miR-452 than sepsis patients without AKI

**Figure 4:**
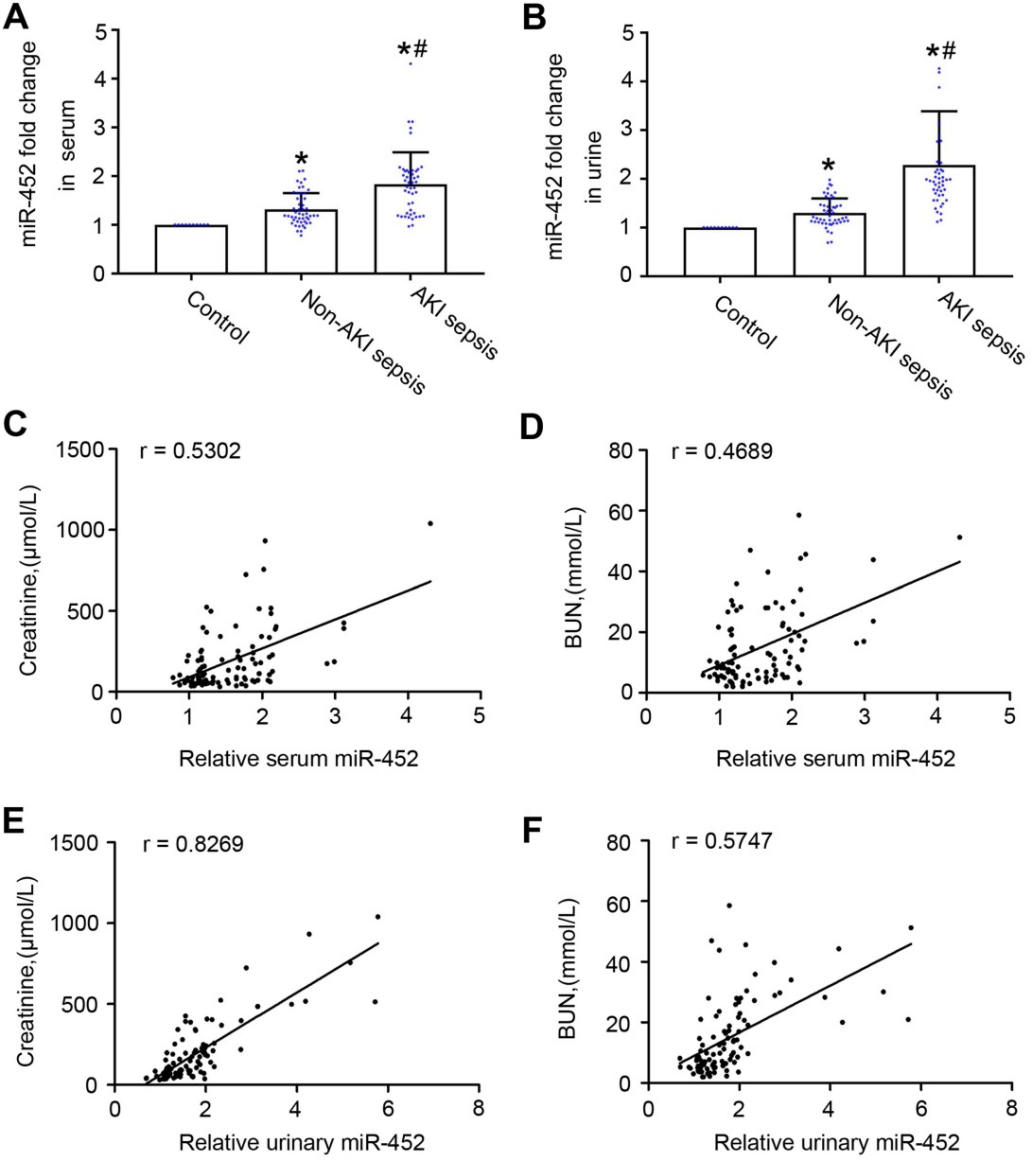
** Sepsis patients with AKI have higher levels of urinary and serum miR-452 than sepsis patients without AKI.** Blood and urine samples were collected from sepsis patients with AKI (AKI sepsis), sepsis patients without AKI (Non-AKI sepsis), and healthy subjects (control) for qPCR analysis of miR-452. miR-452 was normalized with the level of cel-miR-39 (exogenous control added to the samples) to determine the ratios with the ratios of control arbitrarily set as 1. (A) Serum miR-452 in sepsis patients with AKI, sepsis patients without AKI, and healthy controls. (B) Urinary miR-452 in sepsis patients with AKI, sepsis patients without AKI, and healthy controls. Values are mean ± SD. n = 10 for control group, n = 50 for Non-AKI sepsis group, n = 47 for AKI sepsis group, *P < 0.05 vs. healthy controls, #*P <* 0.05 vs. sepsis patients without AKI. (C) Positive correlation between serum miR-452 and serum creatinine in sepsis patients (r=0.5302, Spearman's correlation test). (D) Positive correlation serum miR-452 and BUN in sepsis patients (r=0.4689, Spearman's correlation test). (E) Positive correlation between urinary miR-452 and serum creatinine in sepsis patients (r=0.8269, Spearman's correlation test). (F) Positive correlation between urinary miR-452 and BUN in sepsis patients (r=0.5747, Spearman's correlation test).

To further evaluate the biomarker potential of miR-452, we compared human sepsis patients with AKI to those without AKI for their serum and urinary miR-452. Total of 97 sepsis patients were recruited, including 47 with AKI and 50 without AKI. The control group had 10 healthy people. The clinical information of the participants are shown in Table 1. There were no significant differences between sepsis patient with AKI and those without AKI with respect to age, sex, serum albumin, hemoglobin and Blood sedimentation. As expected, sepsis patient with AKI exhibited significantly higher levels of serum creatinine and BUN, and inflammatory factors (calcitonin, neutrophils, and CRP) than sepsis patients without AKI. qPCR analysis showed higher levels of urinary and serum miR-452 in sepsis patients than healthy control subjects. Importantly, sepsis patients with AKI had significantly higher levels of urinary and serum miR-452 than the patients without AKI (Figure 4A-B). In addition, Spearman's test showed a positive correlation of serum and urinary miR-452 with serum creatinine and BUN in sepsis patients (Figure 4C-F). Of note, the correlation between urinary miR-452 and serum creatinine (Figure 4E: r=0.8269) was markedly better than serum miR-452.

### Serum and urinary miR-452 has high diagnostic efficiency for AKI in sepsis patients

**Figure 5:**
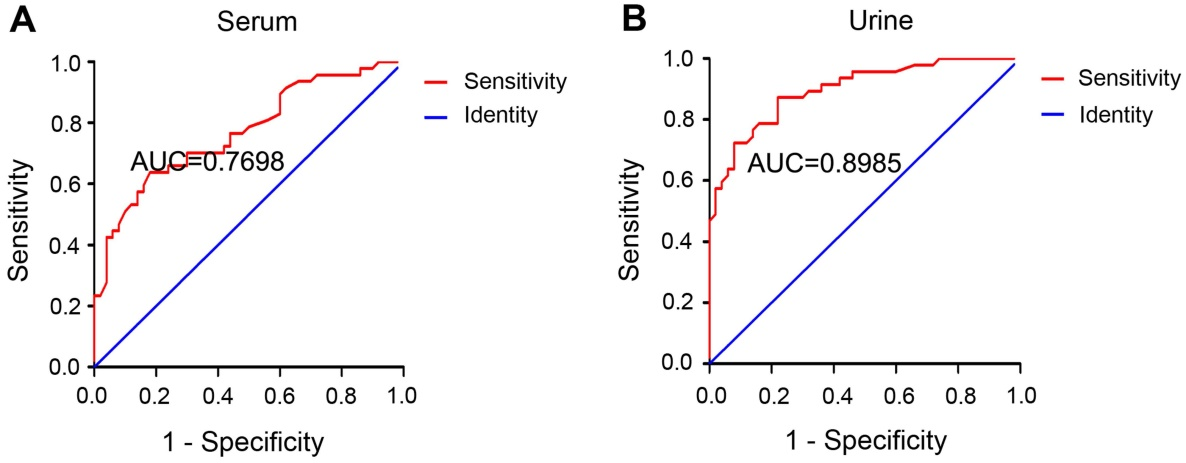
** Serum and urinary miR-452 has high diagnostic efficiency for septic AKI.** ROC curves were plotted by using true positive rate (sensitivity) against the false positive rate (specificity) at different possible cut-points for serum and urinary miR-452 in AKI detection of sepsis patients. The areas under the ROC curves were calculated to show the values of AUC. (A) ROC curve and AUC for the detection of septic AKI using serum miR-452. (B) ROC curve and AUC for the detection of septic AKI using urinary miR-452.

The diagnostic ability of a biomarker is commonly evaluated by the receiver operating characteristic (ROC) curve, where a bigger area under the ROC curve (AUC) indicates higher diagnostic accuracy 40. The AUC was 0.7698 for serum miR-452 (Figure 5A) and 0.8985 for urinary miR-452 (Figure 5B), suggesting a high efficiency of serum and urinary miR-452 in detecting AKI in sepsis patients. The Youden Index (J) calculated as [Sensitivity + Specificity - 1] is a summary statistic of the ROC curve that defines the potential effectiveness of a biomarker. Generally, the maximal Youden Index determines the optimal diagnosis cut-point 40. In our analysis, the optimal cut-point was determined to be 1.66-fold for serum miR-452 and 1.48-fold for urinary miR-452. Based on the optimal cut-points, we calculated that serum miR-452 had a sensitivity of 63.83% and specificity of 82.00%, whereas urinary miR-452 had a sensitivity of 87.23% and specificity of 78.00% for AKI detection in sepsis patients (Table 2). We further examined the diagnostic efficiency of serum miR-452 and urinary miR-452 by binary logistic regression analysis, which showed a 4.68-fold increase of the risk of AKI in sepsis patients for every 1-fold increase in the level of serum miR-452 and a striking 72.48-fold increase of Aki risk for every 1-fold increase of urinary miR-452 (Table 3). Collectively, these data indicate that miR-452, especially urinary miR-452, has high diagnostic ability for AKI in sepsis patients.

### Comparison of the diagnostic performance of Urinary miR-452 vs. urinary [TIMP2]* [IGFBP7]

**Figure 6:**
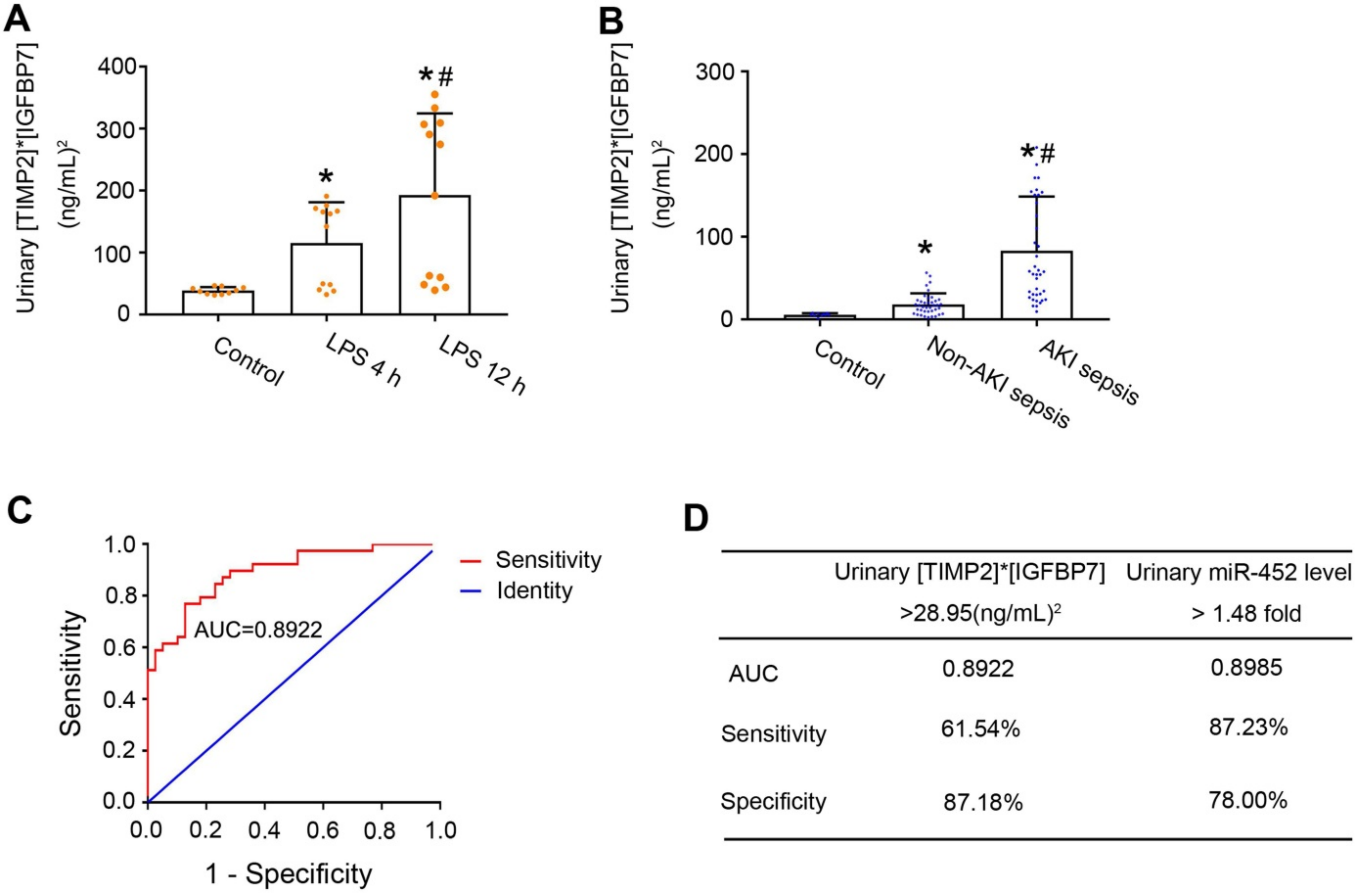
Diagnostic performance of urinary [TIMP2]*[IGFBP7] and its comparison with urinary miR-452.(A) Male C57BL/6 mice were treated with 10 mg/Kg LPS (or saline as control) to collect urine samples at indicated time points to determine TIMP2 and IGFBP7 concentrations by ELISA assay. Values are mean ± SD (n = 10 for each control group, n = 12 for each LPS treatment group), *P < 0.05 vs. control, # *P <* 0.05 vs. LPS 4 h. (B) Urinary [TIMP2]*[IGFBP7] in sepsis patients with AKI, sepsis patients without AKI, and healthy controls. Values are Mean ± SD. n = 6 for control group, n = 39 for Non-AKI sepsis group, n = 39 for AKI sepsis group, *P < 0.05 vs. healthy controls, #*P <* 0.05 vs. sepsis patients without AKI. (C) ROC curve and AUC for the detection of septic AKI using urinary [TIMP2]*[IGFBP7]. (D) Diagnostic performance of urinary [TIMP2]*[IGFBP7] and urinary miR-452 for the detection of septic AKI. The percentage of sepsis patients with AKI detected positive by each biomarker was calculated to show the sensitivity, which was 61.54% (24/39) for [TIMP2]*[IGFBP7] and 87.23% (41/47) for urinary miR-452. The percentage of sepsis patients without AKI detected negative by each biomarker was calculated to show the specificity, which was 87.18% (34/39) for [TIMP2]*[IGFBP7] and 78.00% (39/50) for urinary miR-452.

A major breakthrough in the field of AKI biomarker research is the recent discovery of urinary TIMP2 and IGFBP7 as effective biomarkers for early diagnosis of AKI 17, 18. In 2014, U.S. Food and Drug Administration also approved the marketing of NephroCheck, a test based on the arithmetic result of the concentrations of urinary TIMP2 and IGFBP7 ([TIMP2]*[IGFBP7]), although its diagnostic ability for septic AKI is not optimal 20. We compared urinary miR-452 with urinary [TIMP2]*[IGFBP7] for their diagnostic efficiency in detecting septic AKI in mice and in human patients. Urinary samples were collected to measure the concentrations of TIMP2 and IGFBP7 by ELISA analysis. At both 4 and 12 h after LPS treatment, the mean values of urinary [TIMP2]*[IGFBP7] in mice were significantly bigger than the mean values of control mice (Figure 6A). Of note, LPS-treated mice showed a notable individual variation in their values of urinary [TIMP2]*[IGFBP7] with a subset of mice having values comparable to that of control mice. We further evaluated the urinary [TIMP2]*[IGFBP7] in the sepsis patient cohort. As shown in Figure 6B, sepsis patients with AKI had a significantly bigger value of urinary [TIMP2]*[IGFBP7] than the patients without AKI. Similar to LPS-induced AKI mice, there was a subset of sepsis patients with AKI whose [TIMP2]*[IGFBP7] values were no different from that of patients without AKI. ROC curve analysis showed an AUC of 0.8922 for urinary [TIMP2]*[IGFBP7] (Figure 6C), which was comparable to that of urinary miR-452 (0.8985). The optimal cut-point for [TIMP2]*[IGFBP7] was determined to be 28.95 (ng/mL)^2^. At this cut-point, urinary [TIMP2]*[IGFBP7] had a diagnostic sensitivity of 61.54% and a specificity of 87.18% (Figure 6D). In comparison, urinary miR-452 showed a sensitivity of 87.23% and a specificity of 78% in detecting septic AKI in these patients.

## Discussion

In this study, we have demonstrated miR-452 induction in both *in vitro* and *in vivo* models of septic AKI. The induction was mainly detected in renal tubular cells in kidneys and, mechanistically, it was shown to be mediated by NF-κB. Interestingly, miR-452 also increased in serum and urine of mice at the early phase of septic AKI, prior to detectable kidney injury. Increases of serum and urinary miR-452 were further detected in sepsis patients and, notably, sepsis patients with AKI had significantly higher levels of serum and urinary miR-452 than those without AKI. Compared to the FDA approved AKI biomarker [TIMP2]*[IGFBP7], miR-452, especially urinary miR-452, showed high diagnostic efficiency for AKI in sepsis patients. Thus, miR-452 may be an effective biomarker for early diagnosis of septic AKI.

miR-452 is an evolutionarily conserved microRNA, which has been implicated in a variety of human cancers, including gliomas, colorectal cancer, non-small cell lung cancer, and prostate cancer 41-44. In kidneys, Liu *et al.* showed that miR-452 was up-regulated in clear-cell renal-cell carcinoma where it may promote cell proliferation and invasion by targeting SOX7, contributing to a poor 5-year survival in renal cancer patients 45. This observation was verified by Zhai *et al.*, although miR-452 was suggested to target SMAD4/SMAD7 signaling 46. Other than these two renal cancer studies, there are no reports of miR-452 in kidneys or kidney diseases. In the present study, we have demonstrated the induction of miR-452 in LPS-treated renal tubular cells *in vitro* and in LPS- and CLP-induced septic AKI in mice. Our hybridization analysis further proved the induction in kidney tubular cells in these animals. Consistently, we detected a significant increase of urinary miR-452 in sepsis patients with AKI. These results suggest a role of miR-452 in tubular injury in septic AKI, adding new insights to the complex pathogenesis of this devastating disease 47, 48.

Mechanistically, we have demonstrated the involvement of NF-κB in miR-452 induction in septic AKI. The NF-κB family is composed of five proteins members: p65 (RelA), RelB, c-Rel, p50, and p52 38. Normally, the NF-κB complex is sequestered in the cytoplasm by IκB. Upon stimulation, ΙκΒ is phosphorylated by IκB kinases, ubiquitinylated and degraded, resulting in translocation of the NF-κB complex into the nucleus. Meanwhile, phosphorylation of NF-κB subunits like p65 controls their interactions with other factors, and the stability and transcriptional activity of NF-κB 39. In septic AKI, NF-κB was activated and inhibition of NF-κB could attenuate AKI, indicating a crucial role of NF-κB in the pathogenesis 37, 49. Upon activation, NF-κB drives the transcription and expression of pro-inflammatory cytokines, contributing to Inflammation and associated tissue damage in septic AKI 49, 50. In line with these studies, we detected p65 phosphorylation and the accumulation of phosphorylated p65 (p-p65) in the nucleus of renal tubular cells during LPS treatment *in vitro* and *in vivo* (Figure 2). Moreover, miR-452 induction by LPS in BUMPT cells was abolished by TPCA-1, supporting a critical role of NF-κB in this inductive response. We further identified an NF-κB binding site at the gene promoter of mouse miR-452 and, by ChIP assay, we demonstrated the binding of p65/NF-κB to miR-452 gene promoter sequence during LPS treatment (Figure 2). These results suggest that NF-κB is activated in renal tubular cells during septic AKI to promote miR-452 expression transcriptionally. It remains unclear how miR-452 participates in septic AKI. Nonetheless, we predicted the target genes of miR-452miRNA by using microRNA Target Prediction databases such as TargetScan and miRanda (Table S2). Further investigation may focus on these potential target genes to understand the involvement of miR-452 in septic AKI.

AKI is associated with a remarkably higher rate of mortality in sepsis patients. Thus, early detection of AKI in sepsis would enable timely intervention and supportive care, resulting in the improvement of the clinical outcome and reduction of mortality 1-3, 6, 7. Currently, serum creatinine is still the most commonly used parameter for diagnosis of AKI, but the increase of serum creatinine is a delayed response making it impossible for early AKI detection 12. Consistently, a significant increase of serum creatinine was detected at 12 h after LPS injection or CLP in mice in our study. Recent research has led to the discovery of potential protein biomarkers for AKI, such as KIM-1, NGAL, cystatin C, interleukin-18, TIMP2 and IGFBP7. These biomarkers are usually associated with kidney injury or repair and may be used as a complementary to serum creatinine for an earlier and more sensitive detection. However, unlike other types of AKI, septic AKI often does not induce overt tubular cell injury or necrosis. The mild tubular injury in septic AKI leads to a relatively low sensitivity of these protein biomarkers in early AKI detection in sepsis patients 4, 20.

miRNAs represent a new class of diagnostic biomarkers for human diseases, based on the fact that they are stable and easily detectable in body fluids, including serum and urine 26, 51. Although RNA molecules are known to be highly unstable, miRNAs are remarkably stable and resistant to RNase degradation, extreme pH, and freeze-thaw cycles 21, 22. In addition, sequence-based qPCR amplification enables the measurement of very small amounts of miRNAs in a small volume of body fluids with high specificity and sensitivity 23-25. In the present study, we detected significant increases of serum and urinary miR-452 at 4 h after CLP or LPS-induced AKI in mice, well before detectable kidney injury. Moreover, the levels of serum and urinary miR-452 in sepsis patients with AKI were significantly higher than in sepsis patients without AKI. These observations indicate the potential of serum and urinary miR-452 as a novel biomarker for early detection of septic AKI. Notably, compared to urinary [TIMP2]*[IGFBP7] or the FDA approved AKI biomarker, miR-452 showed a remarkable merit in the sensitivity of AKI detection in sepsis. The sensitivity of urinary miR-452 for AKI detection in sepsis patients reached 87.23%, which was remarkably higher than the sensitivity of [TIMP2]*[IGFBP7] (61.54%) examined in the same cohort of sepsis patients. Especially, a significant portion of sepsis patients with AKI had urinary [TIMP2]*[IGFBP7] that was similar to that of sepsis patients without AKI, resulting in the failure of detecting AKI in these patients by using urinary [TIMP2]*[IGFBP7] (Figure 6). In contrast, compared to sepsis patients without AKI, most patients with AKI had significantly higher levels of urinary miR-452 and were detected by this microRNA. Sensitivity is critical for biomarkers of septic AKI, because kidney tubule injury in this disease is less pronounced than in other types of AKI, and missed diagnosis would cause serious consequences, including prolonged hospital stay and increased morbidity. In this regard, urinary miR-452 has a remarkable advantage, although its specificity (78%) is comparable to or slightly lower than that of [TIMP2]*[IGFBP7] (87.18%).

Recent studies showed that serum and urinary miRNAs may be effective biomarker for prognosis in kidney diseases. For instance, Fourdinier *et al.* demonstrated that lower serum c levels of miR-126 and miR-223 were associated with lower eGFR through studying serum miR-223 and miR-126 levels with clinical outcomes in 601 CKD patients in 6-year follow-up period 52. Lorenzen *et al.* identified plasma miR-210 as a strong and independent predictor of survival in critically ill patients with AKI through analyzing 77 patients with AKI and 30 healthy controls 53. These studies indicate that serum or urinary miRNAs can be effective biomarkers for prognosis and therapeutic efficacy in kidney diseases. The potential of miR-452 as an biomarker for prognosis and therapeutic efficacy of septic AKI needs be determined in future studies.

There are several caveats in this study. First, this is a single center study with a relatively small sample size. It is important to further determine the diagnostic efficiency of miR-452 for septic AKI in larger cohort, multiple center studies. Second, we did not determine the levels of serum and urinary miR-452 during the treatment and recovery of the septic AKI patients. Thus, it remains unclear whether miR-452 may be an effective biomarker for prognosis and therapeutic efficacy. Third, there are reports about miR-452 upregulation in human cancers, raising question about the specificity of miR-452 as a biomarker. In this regard, it is noteworthy that our biomarker work was focused the detection of AKI in sepsis patients and not in the general population. NF-κB regulates the expression of various target genes involved in different pathogenic processes including inflammation. But it is also noteworthy that gene regulation by NF-κB depends on the disease conditions and/or cell types. In the present study, NF-κB mediates miR-452 induction in renal tubular cells in septic AKI. Whether NF-κB activation leads to miR-452 expression in other disease conditions remains unclear. Thus, further research needs to validate the specificity of miR-452 as a biomarker in other diseases. Nonetheless, our study has demonstrated NF-kB-dependent miR-452 induction in renal tubular cells in septic AKI, and has validated its high sensitivity and efficacy in early detection of AKI in sepsis patients.

## Supplementary Material

Supplementary figures and tables.

## Figures and Tables

**Table 1:** Summary of biochemical test results of the participants

	Control	Non-AKI sepsis	AKI sepsis	*P* value
Gender (male/female)	4/6	30/20	20/27	0.09
Age (Years)	55.5±5.8	55.36 ± 2.30	58.72 ± 1.90	0.27
Serum creatinine (µmol/L)	64.34±11.27	70.47 ± 3.81	319.9 ± 30.61	<0.01*
Blood urea nitrogen (mmol/L)	4.15±1.72	5.99 ± 0.36	24.47 ± 1.75	<0.01*
Serum albumin (g/L)	46.05 ± 2.4	27.39 ± 0.93	27.27 ± 0.72	0.92
Hemoglobin (g/L)	122.1 ± 26.8	104.8 ± 2.97	96.36 ± 3.48	0.06
Neutrophils (X10^9^)	3.34 ± 1.46	10.26 ± 0.70	14.14 ± 1.42	0.01 *
Blood sedimentation	-	70.29 ± 4.82,	63.53 ± 5.76	0.37
CRP (mg/L)	-	127 ± 11.92	189.5 ± 20.62	0.01 *
Calcitonin original (ng/L)	-	10.3 ± 3.43	24.15 ± 4.23	0.02 *

Values are mean ± SEM. * significant difference between AKI sepsis patients and Non-AKI sepsis patients.

**Table 2:** Sensitivity and specificity of serum and urinary miR-452 for AKI diagnosis in sepsis patients

	Case	Serum miR-452 level > 1.66 fold	Urinary miR-452 level > 1.48 fold
control	10	0	0
Non-AKI sepsis	50	9	11
AKI sepsis	47	30	41
sensitivity		63.83%	87.23%
specificity		82.0%	78.0%

**Table 3:** Binary logistic regression analysis of serum miR-452 and urinary miR-452 for AKI diagnosis in sepsis patients

	Wald	df	Significance	Odds Ratio	95% CI for Odds Ratio	
					Lower	Upper
Serum miR-452	4.99	1	0.025	4.68	1.21	18.10
Urinary miR-452	20.03	1	<0.001	72.48	11.11	472.93

The results of using serum and urinary miR-452 for AKI detection in sepsis patients were analyzed by binary logistic regression. The logistic model was statistically significant (χ^2^ = 63.97, *P <* 0.001). The model suggested a 4.68-fold increase of the risk of AKI in sepsis patients for every 1-fold increase in serum miR-452 and a 72.48-fold increase for every 1-fold increase of urinary miR-452.

## Abbreviations

AKI: Acute Kidney Injury
BUMPT: The Boston University mouse proximal tubular cell line
CLP: cecal ligation and puncture
IGFBP7: insulin-like growth factor binding protein 7
KIM-1: kidney injury molecule-1
LPS: lipopolysaccharides
L-FABP: liver fatty acid-binding protein
miRNA: MicroRNA
NGAL: neutrophil gelatinase-associated lipocalin
NF-κB: nuclear factor kappa B
ROC: Receiver operating characteristic
TIMP2: tissue inhibitor of metalloproteinase 2
