# Supplementary material for: Discovery and validation of miR-452 as an effective biomarker for acute kidney injury in sepsis
Source: Theranostics. 2020 Oct 25;10(26):11963–75. doi: 10.7150/thno.50093 (PMC7667674; doi:10.7150/thno.50093)
Supplement: Supplementary file 1 — Supplementary figures and tables. [file thnov10p11963s1.pdf]

Discovery and validation of miR-452 as an effective biomarker for acute kidney injury in sepsis

Zhiwen Liu[1], Danyi Yang[1], Jingli Gao[1], Xiaohong Xiang[1], Xiaoru Hu[1], Siyao Li[1], Wenwen Wu[1], Juan Cai[1], Chengyuan Tang[1], Dongshan Zhang[2], Zheng Dong[1,3] *

Supplementary Data

2 Supplementary Figures

2 Supplementary Tables

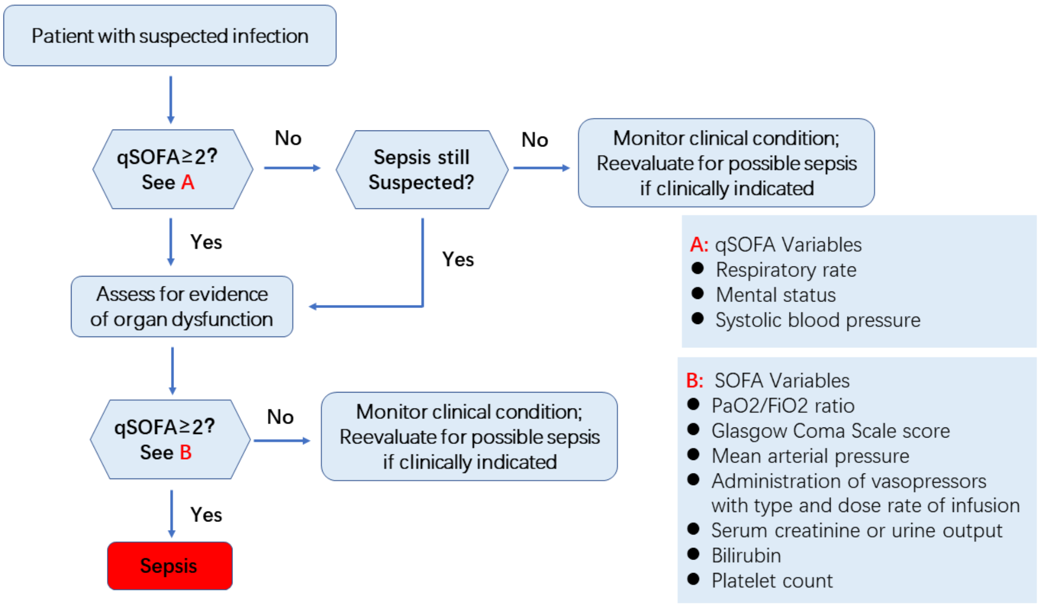

Figure S1. Procedure for clinical diagnosis of patients with sepsis. The baseline Sequential [Sepsis-related] Organ Failure Assessment (SOFA) score is zero unless the patient is known to have preexisting (acute or chronic) organ dysfunction before the onset of infection. qSOFA: quick SOFA.

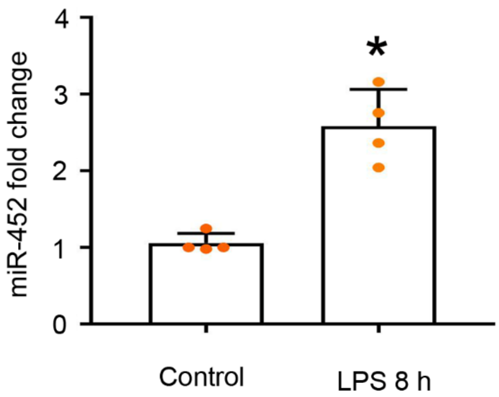

Figure S2. Induction of miR-452 by LPS in BUMPT cells. BUMPT cells were maintained in DMEM containing 0.2% FBS for 24 h followed by treatment with 10 µg/mL LPS in DMEM containing 0.2% FBS for 8 h. miR-452 was quantified by Taqman assay-based qPCR and normalized to the level of sno202 (internal control) to determine the ratios with the ratios of control cells arbitrarily set as 1. Values are mean ± SD, *P<0.05 vs. control cells.

Table S1. AKI diagnosis according to KIDIGO guidelines.

Stage	Serum creatinine	Urine output
1	1.5–1.9 times baseline<br>or<br>⩾0.3 mg/dL (⩾26.5 mmol/L) Increase	<0.5 mL/Kg/h for 6–12 h
2	2.0–2.9 times baseline	<0.5 mL/Kg /h for ⩾12 h
3	3.0 times baseline<br>or<br>Increase in serum creatinine level to⩾4.0 mg/dL (⩾353.6 mmol/L)<br>or<br>Initiation of renal replacement therapy<br>or<br>In patients younger than 18, decrease in eGFR to <35 mL/min per 1.73 m^2	<0.3 mL/Kg/h for 24 h<br>or<br>Anuria for ⩾12 h

AKI, acute kidney injury; eGFR, estimated glomerular filtration rate.

Table S2: Predicted targets of mmu-miR-452

Gene name	Numbers of supported Ago CLIP-Seq experiments	PITA	RNA22	miRmap	microT	miRanda	PicTar	TargetScan
Ldlr	8	1	0	1	0	0	0	0
Acsl3	6	1	0	1	0	0	0	0
Lefty1	6	1	0	1	1	0	0	0
Ankrd52	6	1	0	1	1	0	0	0
Kpnb1	6	1	0	0	0	0	0	0
Sel1l	6	1	0	0	1	0	0	0
Polr3g	6	1	0	0	0	0	0	0
Ifngr2	6	1	0	0	0	0	0	0
Klf3	6	1	0	0	0	0	0	0
Rab8b	6	1	0	0	1	0	0	0
Wdr26	5	1	0	1	2	0	0	0
Lefty1	5	1	0	1	1	0	0	0
Apaf1	5	1	0	1	1	0	0	0
Mat2b	5	1	0	0	0	0	0	0
Itm2b	5	1	0	0	0	0	0	0
Zbtb43	5	1	0	0	0	0	0	0
Spp1	5	1	0	1	1	0	0	0
Arl6ip5	5	1	0	1	1	0	0	0
Asah1	5	1	0	0	0	0	0	0
Otud4	5	1	0	1	0	0	0	0
Zc3h12c	5	0	0	1	0	0	0	0
